# The Gut Microbial Community of Antarctic Fish Detected by 16S rRNA Gene Sequence Analysis

**DOI:** 10.1155/2016/3241529

**Published:** 2016-11-13

**Authors:** Wei Song, Lingzhi Li, Hongliang Huang, Keji Jiang, Fengying Zhang, Xuezhong Chen, Ming Zhao, Lingbo Ma

**Affiliations:** Key Laboratory of East China Sea and Oceanic Fishery Resources Exploitation, East China Sea Fisheries Research Institute, Chinese Academy of Fishery Sciences, Ministry of Agriculture, Shanghai, China

## Abstract

Intestinal bacterial communities are highly relevant to the digestion, nutrition, growth, reproduction, and a range of fitness in fish, but little is known about the gut microbial community in Antarctic fish. In this study, the composition of intestinal microbial community in four species of Antarctic fish was detected based on 16S rRNA gene sequencing. As a result, 1 004 639 sequences were obtained from 13 samples identified into 36 phyla and 804 genera, in which Proteobacteria, Actinobacteria, Firmicutes, Thermi, and Bacteroidetes were the dominant phyla, and* Rhodococcus*,* Thermus*,* Acinetobacter*,* Propionibacterium*,* Streptococcus*, and* Mycoplasma* were the dominant genera. The number of common OTUs (operational taxonomic units) varied from 346 to 768, while unique OTUs varied from 84 to 694 in the four species of Antarctic fish. Moreover, intestinal bacterial communities in individuals of each species were not really similar, and those in the four species were not absolutely different, suggesting that bacterial communities might influence the physiological characteristics of Antarctic fish, and the common bacterial communities might contribute to the fish survival ability in extreme Antarctic environment, while the different ones were related to the living habits. All of these results could offer certain information for the future study of Antarctic fish physiological characteristics.

## 1. Introduction

Although the Southern Ocean occupies 10% of the world's ocean, only 322 species fishes in Antarctic Ocean were recognized currently, considered so small comparing to the global diversity approximately 25,000~28,000 species, while the benthic fish fauna includes 19 families of about 222 species [1].

Antarctic fishes have been isolated for over 10 million years. Besides, they have developed mechanisms to adapt to and survive in the coldest and most thermally stable environment now [2–5]. Antarctic water under the sea ice possesses a very low and fairly constant temperature of about −1.86°C, and annual temperature fluctuations are in 1°C; Antarctic sea water is with a high oxygen concentration of 0.18–0.36 mmol/L, which enables ice fish to live with no haemoglobin [6–8].

*Trematomus bernacchii* (family Notothenioidei),* Chionodraco hamatus*, (family Channichthyidae),* Gymnodraco acuticeps* (family Bathydraconidae), and* Pagothenia borchgrevinki* (family Notothenioidei) are four Antarctic fish living in the oxygen-rich coastal Antarctic Ocean with the equilibrium temperature at −1.86°C year round [2, 9].* T*.* bernacchii*,* C. hamatus*, and* G. acuticeps* are benthic fish and always live in the depth of more than 100 m under the surface of the sea ice, while* P. borchgrevinki* likely live in the water 1~2 m below the ice [10–13].

Fish have stable microbiota in the gastrointestinal (GI) tract, and the microbiota considered as an integral part of the host is highly relevant to the digestion, nutrition, growth, and reproduction and strongly affects fish health by assisting the gut epithelium development and stimulating the innate immune system [14, 15]. Furthermore, some papers have provided that the microbes living in the fish intestines are influenced by dietary manipulations [16] while little is known about the bacterial community composition of Antarctic fish and whether the Antarctic fish gut bacterial community was affected by extreme environmental conditions.

16S rRNA-based molecular methodologies are now commonly used for identifying and classifying the bacterial species within compounded microbial communities [17]. In this study, we used Illumina MiSeq platform and comparative sequence analysis to determine the microbial diversity of* T. bernacchii*,* C. hamatus*,* G. acuticeps,* and* P. borchgrevinki.*

## 2. Materials and Methods

### 2.1. Sample Collection and Preparation

A total of thirteen fish belonging to four species, four of* T. bernacchii*, two of* C. hamatus*, five of* G. acuticeps,* and two of* P. borchgrevinki, *were used. All of these fish were caught at 100–200 m under the ice by net near the location of 72°55′E and 67°29′S through Chinese Antarctic research vessel Xue Long.

Experimental fish were randomly harvested with net and then euthanized by an overdose of MS 222. After dissection, the intestines were removed aseptically from each fish abdominal cavity; the contents were carefully collected and labelled Tb1–Tb4 for* T. bernacchii*, Ch1–Ch5 for* C. hamatus*, Ga1-Ga2 for* G. acuticeps,* and Pb1-Pb2 for* P. borchgrevinki* and stored at −80°C before transporting to the laboratory.

### 2.2. DNA Extraction

The intestinal content samples were thawed on ice, and then genomic DNA were separately extracted using the E.Z.N.A. Stool DNA kit (OMEGA, Bio-Tek, USA) based on the manufacturer's protocol and stored at −80°C. The integrity of the 13 DNA samples was assessed visually using agarose gel (containing ethidium bromide) electrophoresis on 1.0% and quantified using a Qubit v2.0 fluorometer (Life Technologies, Darmstadt, Germany). The DNA concentration was determined by using a fluorescence spectrophotometer (ES-2, Malcom, Japan).

### 2.3. PCR Amplification and Sequencing

The hypervariable regions V4-V5 of the 16S rRNA gene were amplified using a universal primer set 515 F (5-GTGCCAGCMGCCGCGG-3) and 907 R (5-CCGTCAATTCMTTTRAGTTT-3). The PCRs were performed in triplicate using 25 *μ*L reaction system with 1 *μ*L each primer (10 *μ*M), 2 *μ*L DNA template (20 ng/*μ*L), 5 *μ*L 5x Q5 reaction buffer, 5 *μ*L 5x Q5 GC high enhancer, 2 *μ*L dNTPs (2.5 mM), and 0.25 *μ*L Q5 polymerase (5 U/*μ*l). The PCR amplification conditions were 1 cycle of 98°C for 3 min (initial denaturation), followed by 25 cycles of 98°C for 15 s (denaturing), 50°C for 30 s (annealing) and 72°C for 30 s (extension), and finally 1 cycle of 72°C for 5 min (final extension). The amplified PCR products were examined by 2% gel electrophoresis, purified by using the MinElute Gel Extraction Kit (Qiagen) to remove the unspecific DNA fragments and quantitated by using Bioanalyzer 2100 (Agilent Technologies, Waldbronn, Germany). The products were pooled together with equal amount and sequenced on the Illumina MiSeq platform (Roche Applied Science, Indianapolis, IN, USA). And the length of paired-end reads was 150 bp.

### 2.4. Data and Statistical Analysis

The raw sequences obtained from Illumina MiSeq were firstly filtered for quality control and reads with length <150 bp, ambiguous bases, average base quality score of <20 in the tags were discarded. Then FLASH (version 1.2.7, http://ccb.jhu.edu/software/FLASH/) was used to merge read1 and read2 [18], and sequences with overlap <10 and mismatches were removed. After that, Quantitative Insights Into Microbial Ecology (QIIME) (version 1.9.0, http://qiime.org/) [19] was used to the further quality control and uchime of mothur (version 1.31.2) [20, 21] was used for chimera checking.

Reads after quality control were delineated into operational taxonomic units (OTUs) with a 97% sequence similarity using uclust of QIIME, and OTUs with abundance less than 0.001% of the total sequences were discarded. The taxonomic information of the representative sequence in each OTU was obtained by matching sequence database using BLAST of QIIME.

The rarefaction curves and bar graph of species distribution for the 13 samples were constructed using QIIME and the alpha-diversity indices (i.e., Chao1 estimator and Shannon estimator) were calculated using mothur. The analysis of shared and unique operational taxonomy unit (OTU) between the four species was conducted based on the OTU table generated by the QIIME (v1.9.0).

To compare the similarity of the microbial community composition among the 13 intestinal contents of the four species of Antarctic fish, difference of microbial community in each sample was calculated by the Principal Components Analysis (PCA) and the heatmap associated with evolutionary relationship among different samples was also constructed and analyzed.

## 3. Results

### 3.1. The Microbial Complexity

A total of 1 061 710 sequences were obtained from 13 samples with the number of sequences ranging from 28 296 to 138 254 per individual after filtering for quality. By removing chimeras, 26 978 to 119 888 sequences were collected from each sample, resulting in a total of 1 004 639 sequences from all samples. Then all the sequences were clustered into 2199 OTUs at the 97% sequence similarity value (Table 1).

The microbial complexities in the gut of* T. bernacchii*,* C. hamatus*,* G. acuticeps,* and* P. borchgrevinki* were estimated on the basis of alpha-diversity indices (Chao1 indices and Shannon indices). The Chao1 was used to estimate species richness, while Shannon's index was used to indicate species diversity. The results showed that* C. hamatus* samples had the largest alpha-diversity indices followed by* G. acuticeps, T. bernacchii,* and* P. borchgrevinki* (Table 2).

### 3.2. Microbial Community Composition

**Figure 1:**
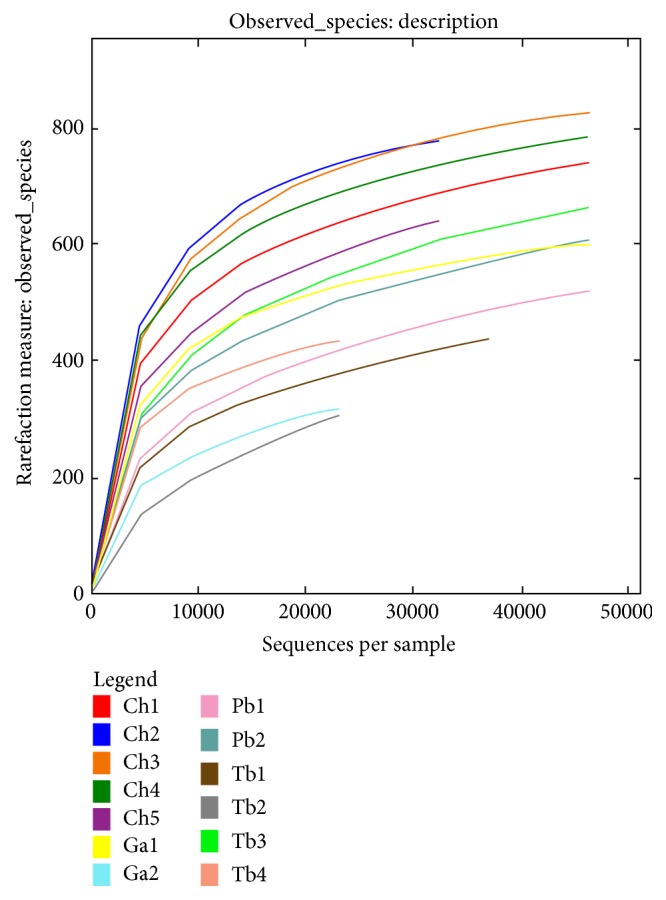
Rarefaction curve.

After sampling 20000 reads, with the sampled read number increasing, the newly discovered OTUs reduced and the rarefaction curves tended to attain the saturation plateau (Figure 1). This showed that the libraries of the 13 samples were large enough to estimate the phylotype richness and microbial community diversity at the 97% similarity threshold.

**Figure 2:**
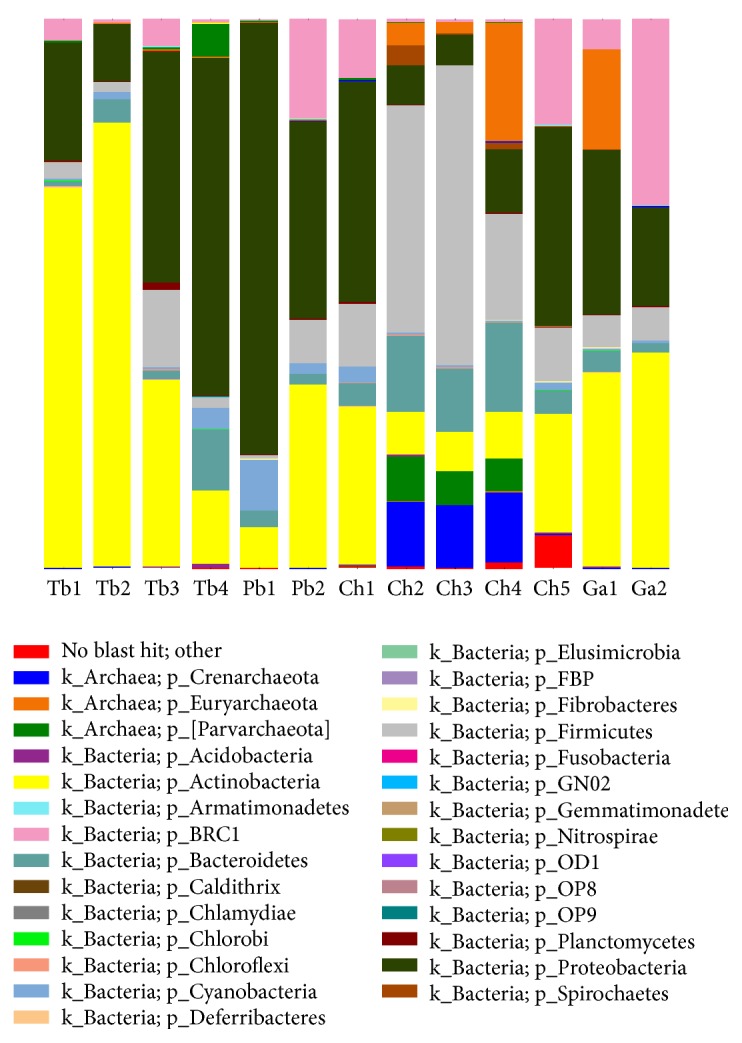
The bacterial community composition in different samples.

All sequences were identified into 36 phyla, and only 0.6% of the total sequences were assigned to unspecified microbial phyla. Phyla with abundance >0.1% of the 13 samples were clearly observed in the bar graph of species distribution (Figure 2). Proteobacteria (30.8%), Actinobacteria (29.8%), Firmicutes (13.7%), Thermi (7.6%), and Bacteroidetes (6%) were the most dominant groups which accounted for 87.90% of the reads and commonly observed in all 13 fish guts. Other major phyla, including Tenericutes (3.6%), Crenarchaeota (2.8%), and Cyanobacteria (1.8%), were also identified in all fish samples, but Crenarchaeota, Parvarchaeota (1.6%), and Euryarchaeota were the only three phyla belonging to Archaea, and the latter two were only present in* Chionodraco hamatus*. Though the major bacterial phyla in the 13-fish intestinal content were similar, the relative abundance was obviously different.

At the genus level, the sequences from 13 samples were identified into 804 genera ranging from 102 to 210 per individual. The gut content samples were dominated by six major genera, representing approximately 49.3% of the sequences, including* Rhodococcus* (19.5%),* Thermus* (7.5%),* Acinetobacter* (7.1%),* Propionibacterium* (6.5%),* Streptococcus* (5.1%), and* Mycoplasma* (3.6%). All above genus and Corynebacterium (1.8%) and Flavobacterium (1.3%) were present in all intestinal content samples.

### 3.3. Common and Unique Microbial Communities

The analysis of the common and unique OTUs was conducted to investigate the gut microbial community in different fish through a Venn diagram. Pairwise comparison was performed among the four species fish via considering the shared OTUs, as those present in a certain percentage at least 30% or 40% of the samples of each species fish gut, and the unique OTUs were defined as those only present in more than 30% or 40% of the samples taken from one species of fish gut sample and unfound in the other three species of fish gut samples.

**Figure 3:**
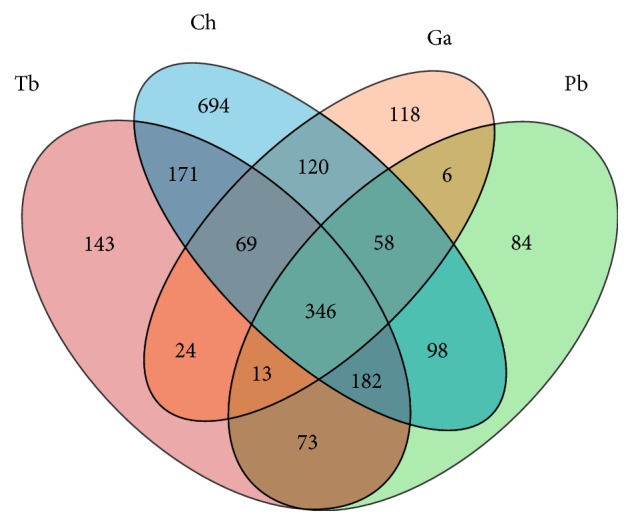
The numbers of common and unique OTUs presented in the four species of Antarctic fish.

The number of common OTUs ranged from 346 to 768 and unique OTUs varied from 84 to 694 (Figure 3). Many OTUs were uniquely present in only one species of fish gut sample, especially in* T. bernacchii* (about 694), while the common OTUs in the four species of fish gut were not a few yet (over 346).

### 3.4. The Similarity of Microbial Community Composition

**Figure 4:**
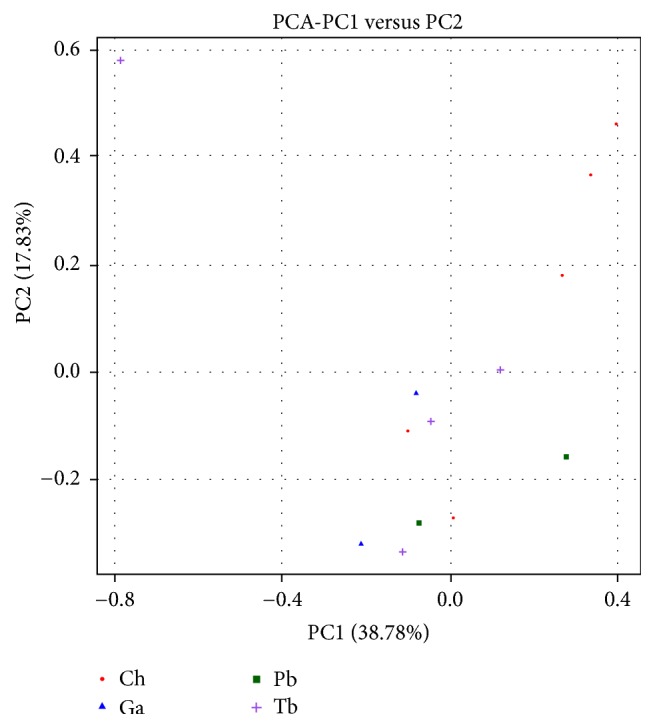
Principal Components Analysis (PCA) of the dissimilarity between the microbial samples.

The similarity and difference of microbial community compositions of 13 intestinal content samples taken from the four species of Antarctic fish* (T. bernacchii*,* C. hamatus*,* G. acuticeps,* and* P. borchgrevinki)* were showed in PCA plot with PC1 accounting for 38.78% of the total variation and PC2 for 17.83%. As a result, no species, except 3* C. hamatus* samples and 1* T. bernacchii* sample, formed a distinct cluster and clearly separated from other three species (Figure 4).

**Figure 5:**
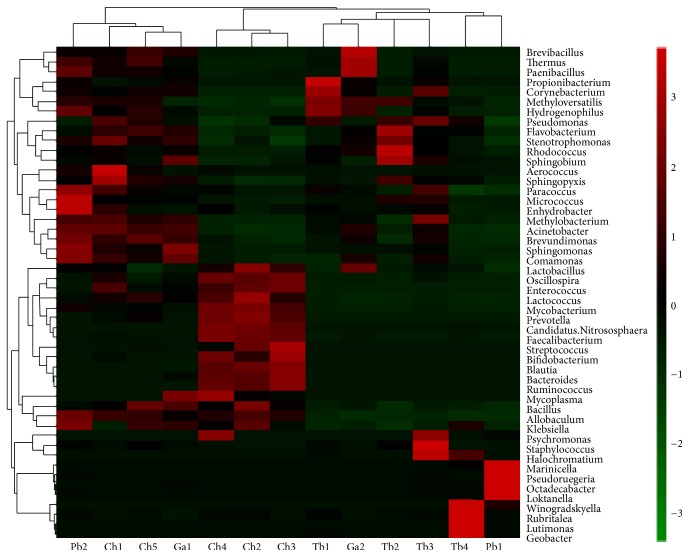
Microbial distribution of different samples.

The hierarchically clustered heatmap analysis associated with the similarity of microbial community composition was performed at the genus level disclosing the richness and diversity of bacterial communities in the gut content of each sample. The composition of intestine microbiota could not show obvious similarity based on each species of Antarctic fish, but for individual, Ph2, Ch1, Ch5, and Ga1 showed higher similar, Ch2, Ch3, and Ch4 had higher similarity, Tb1, Tb2, Tb3, and Ga2 had closer relationship, and Tb4 and Pb1 showed higher similarity (Figure 5).

## 4. Discussion

As is well known, the habitat is a key factor for the survival of organisms, and living temperature represents a significant driving force for biological evolution. Evolution of Southern Ocean organism occurred accompanied by the striking albeit intermittent temperature for about 60 million years [22]. The habitat modifications force the fish fauna to develop a number of morphological and physiological adaptations in order to survive in a cold, highly oxygenated environment without hematocrit and hemoglobin [23, 24].

*T. bernacchii*,* C. hamatus*,* G. acuticeps,* and* P. borchgrevinki* are four order Perciformes and important species in the Antarctic Ocean. Although studies on fish intestinal microbiota have been reported and the mechanisms of the survival ability about Antarctic fish have been shown in many papers, little is still known about the intestinal microbial community in Antarctic fish [25–29].

This study aims to detect the composition of intestinal microbial community in four species of Antarctic fish based on 16S rRNA gene sequence through Illumina MiSeq platform. As a result, 1 004 639 sequences were obtained and clustered into 2199 OTUs based on 97% sequence similarity level and identified into 36 phyla and 804 genera for the 13 samples, showing a large microbial diversity in the Antarctic fish. Actinobacteria, Proteobacteria, Firmicutes, Thermi, and Bacteroidetes were the most dominant groups at phylum level, and* Rhodococcus*,* Thermus*,* Acinetobacter*,* Propionibacterium*,* Streptococcus,* and* Mycoplasma* were more abundant in the genus level. The result that Proteobacteria and Firmicutes act as dominant groups at phylum level is consistent with the previous finding [29]. Firmicutes and Bacteroidetes contribute to carbohydrates and/or proteins fermentation in the intestine to help the host acquire nutrients from the diet [30]. Crenarchaeota was presented in the four species and accounted for quite a proportion, which is similar to Wilkins et al. [31] study about shaping factors of Southern Ocean microbial assemblage, and this might suggest that Crenarchaeota is related to Antarctic environment. In addition, Actinobacteria and Gammaproteobacteria were two highly abundant classes accounting for 29.4% and 16.4% of total dataset, respectively, which is in accordance with the result of Mosier Annika et al. study that Actinobacteria (42%) dominated in the surface ice community and Gammaproteobacteria (52%) dominated in the deep ice community [32].

The Venn diagram showed that the four species of Antarctic fish shared many OTUs and also each of them had many unique OTUs which indicates that some similar microbiota lives in intestine of the four species because of the same living conditions, while different organisms are parasitic in the gut for the reason of different life habits and species. The PCA and heatmap presented no obvious difference or similarity in the intestinal microbial community composition among the four species, and we are speculating that bacterial communities in Antarctic fish intestinal tract might have an influence on the physiology of digestion as at present there is no evidence for it.

## 5. Conclusion

The diet and the environment affect the intestinal microbiota of fish and mammals [33, 34]. In the present study, individuals of each species harbored not really similar intestinal bacterial communities, and gut bacterial communities among the four species were also not absolutely different, suggesting that the common bacterial communities in Antarctic fish intestinal tract might contribute to the fish survival ability in extreme Antarctic environment, while the different bacterial communities might be related to the living habits such as diet, depth, and location. All of these results may contribute to the further study of the relationship between the intestinal bacterial communities and physiological characteristics of Antarctic fish.

## Figures and Tables

**Table 1:** Sequence and taxonomy number of individual sample.

Samples	Effective sequence	High quality sequence	Phylum	Class	Order	Family	Genus
Tb1	39021	37534	433	432	427	373	236
Tb2	28296	27392	321	321	318	276	163
Tb3	52250	49982	662	659	652	581	352
Tb4	28698	26978	449	447	440	381	190
Pb1	53101	49930	527	525	514	452	249
Pb2	61641	59218	642	640	634	571	375
Ch1	49882	47539	719	717	698	612	413
Ch2	39837	36146	776	776	768	616	377
Ch3	75228	71669	865	863	855	677	401
Ch4	64234	60660	798	796	788	633	379
Ch5	40188	37524	616	614	606	538	350
Ga1	59050	57647	616	616	607	521	338
Ga2	29089	27200	335	335	333	301	207
Total	1061710	1004639					

**Table 2:** The alpha indices of different samples.

Samples	Chao1^a^	ACE	Simpson	Shannon
Tb1	680.5789	590.8154	0.782606	3.728343
Tb2	593.1923	717.972	0.405413	2.07083
Tb3	862.28	827.5564	0.928014	5.277833
Tb4	664.5172	542.6397	0.955801	5.769862
Pb1	845.8936	734.8494	0.905419	4.801013
Pb2	890.375	847.3593	0.910085	5.060302
Ch1	908.3	867.9615	0.918179	5.479842
Ch2	891.25	850.9911	0.941227	6.02579
Ch3	1004	948.9715	0.894694	5.562919
Ch4	983.7581	927.8588	0.934068	6.029751
Ch5	886.8971	848.5095	0.927018	5.530419
Ga1	756.8333	706.2764	0.892472	4.79657
Ga2	551.7576	504.3151	0.800829	3.598119
